# The Adenomatous Polyposis Coli Protein Contributes to Normal Compaction of Mitotic Chromatin

**DOI:** 10.1371/journal.pone.0038102

**Published:** 2012-06-13

**Authors:** Dina Dikovskaya, Guennadi Khoudoli, Ian P. Newton, Gaganmeet S. Chadha, Daniel Klotz, Ashwat Visvanathan, Angus Lamond, Jason R. Swedlow, Inke S. Näthke

**Affiliations:** 1 Cell and Developmental Biology, University of Dundee, Dundee, Scotland, United Kingdom; 2 Wellcome Trust Centre for Gene Regulation and Expression, University of Dundee, Dundee, Scotland, United Kingdom; University of Edinburgh, United Kingdom

## Abstract

The tumour suppressor *Adenomatous Polyposis Coli* (APC) is required for proper mitosis; however, the exact role of APC in mitosis is not understood. Using demembranated sperm chromatin exposed to meiotic *Xenopus egg* extract and HeLa cells expressing fluorescently labelled histones, we established that APC contributes to chromatin compaction. Sperm chromatin in APC-depleted *Xenopus* egg extract frequently formed tight round or elongated structures. Such abnormally compacted chromatin predominantly formed spindles with low microtubule content. Furthermore, in mitotic HeLa cells expressing GFP- and mCherry-labelled H2B histones, depletion of APC caused a decrease in the donor fluorescence lifetime of neighbouring fluorophores, indicative of excessive chromatin compaction. Profiling the chromatin-associated proteome of sperm chromatin incubated with *Xenopus* egg extracts revealed temporal APC-dependent changes in the abundance of histones, closely mirrored by chromatin-associated Topoisomerase IIa, condensin I complex and Kif4. In the absence of APC these factors initially accumulated on chromatin, but then decreased faster than in controls. We also found and validated significant APC-dependent changes in chromatin modifiers Set-a and Rbbp7. Both were decreased on chromatin in APC-depleted extract; in addition, the kinetics of association of Set-a with chromatin was altered in the absence of APC.

## Introduction

The tumor suppressor Adenomatous Polyposis Coli (APC) is a large multifunctional multi-domain protein that regulates beta-catenin turnover and also organizes the microtubule cytoskeleton [1]. Mutations in APC are causal to colorectal cancers. Intestinal crypt cells lacking functional APC are hyperproliferative and display differentiation and migration defects [2]–[3]. Furthermore, loss of APC correlates with chromosomal instability (CIN) in cancers and induces CIN in cultured cells and mouse models [4]. While some of these phenotypes are mediated by the enhanced beta-catenin signaling that results from the absence of wild type APC [5]–[6], loss of beta-catenin–independent function(s) of APC contributes to defective mitoses responsible for CIN [7]–[9]. For instance, the ability of APC to crosslink and stabilise microtubules during spindle formation [10] explains some mitotic defects that occur in the absence of APC.

Chromatin condensation in mitosis and meiosis causes a measurable increase in chromatin compaction [11], [12], [13]. The state of chromatin compaction is an important player in mitosis, as defects in mitotic chromosome architecture correlate with chromosomal mis-segregations [14]. At metaphase, the condensin-dependent rigidity of centrosomes [15] contributes to correction of inappropriate microtubule attachments to kinetochores [16]. In anaphase, further compaction of chromosomes is important for complete segregation of chromosomes [17]–[18].

Here we describe a novel role of APC in chromatin compaction, in two unrelated systems: mitotic HeLa cells and *Xenopus* sperm chromatin remodeled in meiotic *Xenopus* egg extract. Furthermore, we found that in *Xenopus* egg extract, depleting APC results in parallel changes in the kinetics of association of condensin I, Topoisomerase II, Kif4 and linker histones with chromatin during the remodeling process. In addition, loss of APC reduces the association of chromatin modifiers Set-a and Rbbp7 with chromatin in this system.

## Results

### APC Depletion Alters Chromatin Compaction in Xenopus Egg Extracts

APC plays an important role in spindle formation in several experimental systems; however, the exact mechanism by which APC supports spindles is not completely understood. We previously established a requirement for APC in spindle formation in *Xenopus* egg extracts arrested at metaphase of meiosis II (CSF extracts). Spindles formed in such extracts without APC are less robust and have reduced microtubule mass. This is particularly evident in the spindle mid-zone - a phenotype we referred to as “weak spindles” [9], [19].

**Figure 1:**
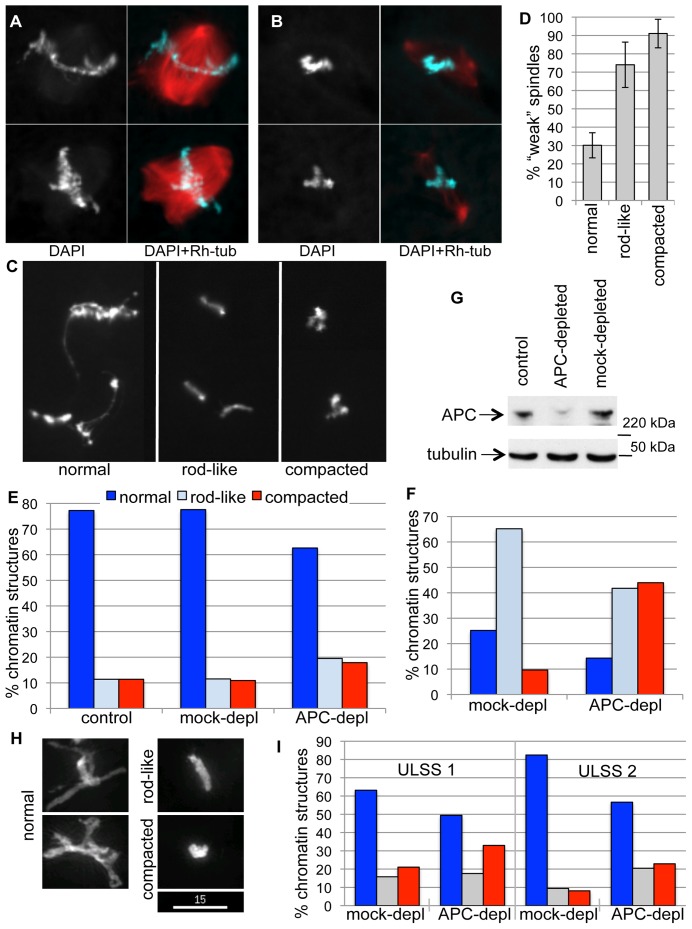
APC depletion changes chromatin appearance in *Xenopus* egg extract. Demembranated sperm chromatin was incubated with CSF extract in the presence of a small amount of Rhodamine-labelled tubulin for 1 h, overlayed with DAPI-containing fixative and imaged. ***A*** *.* Two representative examples of “strong” spindles formed in such extract, with a dense microtubule network and well-resolved, mitotically condensed chromatin. ***B*** *.* Two representative examples of “weak” spindles formed in CSF extract with low microtubule content and abnormally compacted chromatin. ***C*** *.* Typical morphology of chromatin classified here as normal (left panel), rod-like (middle panel) and compacted (right panel) used for the quantification shown in D-F. ***D*** *.* Proportion of phenotypically “weak” spindles formed around chromatin with normal, rod-like and compacted morphology was determined in three different preparations from the same extract. Between 62 and 85 spindles per slide were counted. ***E*** *.* Quantification of chromatin morphology by categories depicted in C, after incubation in untreated (control), mock- or APC-depleted extracts for 1 h. 80–165 chromatin figures were scored for each condition. ***F*** *.* Quantification of different chromatin morphologies according to categories depicted in C, after incubation in mock- or APC-depleted extracts for 1 h, followed by exposure to +12°C. 70–90 chromatin figures were scored for each condition. ***G*** *.* Level of APC depletion in CSF extract visualized by immunoblotting mock- and APC-depleted extracts with anti-APC antibody. Tubulin was used as a loading control. ***H*** *.* Representative images of demembranated sperm chromatin after incubation in ULSS extract at 23.5°C for 50 min, classified as normal, rod-like and compacted for quantification in I. Sum intensity projections of deconvolved z-stack images capturing the total chromatin fluorescence are shown. Size bar 15 µm. ***I*** *.* Percent of normal, rod-like and compacted chromatin (as depicted in H) detected after incubation in mock- or APC-depleted ULSS extract after 40 (ULSS 1) or 50 (ULSS 2) min. Data from two independent experiments are shown. 74–133 chromatin figures were scored for each condition.

The inherent variability of the *Xenopus* extract system [19] allowed us to observe that the more pronounced “weak spindle” phenotype is usually associated with a more compacted chromatin morphology (Fig. 1 A and B). To quantify this observation, we measured the correlation between spindle and chromatin morphology. To this end we classified chromatin into normal (e.g. mitotically condensed, displaying the typical pattern of alternating thin strings and thicker blocks, Fig. 1C, left), rod-like (e.g. without thin strings but retaining an elongated shape, Fig. 1 C middle) and compacted (composed of one or several rounded thick blocks Fig.1 C right). We then assessed spindle phenotype and chromatin morphology in the same spindles and confirmed that abnormally compacted chromatin was three times more likely to associate with weak spindles (Fig. 1 D).

Since APC depletion significantly increased the proportion of weak spindles formed in CSF extracts [19], we asked whether it also led to a change in chromatin compaction. To quantify the effect of APC removal on chromatin morphology, we analysed chromatin structures in untreated (control), APC- and mock-depleted CSF extracts (Fig. 1E, G) supplemented with *Xenopus* sperm-derived chromatin. Chromatin was incubated in an extract for 55 min at room temperature, sufficient time to complete spindle formation. APC depletion almost doubled the fraction of rod-like and compacted chromatin in comparison to control or mock–depleted extract (Fig. 1 E) confirming that chromatin compaction was altered in APC–depleted extracts.

APC stabilizes microtubules, and lack of APC in cells causes reduced tension on metaphase kinetochores [8]–[9]. It is therefore possible that alteration in morphology of mitotic chromatin in the absence of APC is due to reduced pulling forces from spindle microtubules. However, the effect of APC depletion on chromatin compaction was even more apparent when microtubules were de-polymerised by exposing the extract to cold temperature (Fig. 1 F, G), suggesting that the effect of APC on chromatin compaction does not require microtubules.

We also considered the possibility that the compacted chromatin structures frequently detected in APC–depleted extracts represented fragments of chromatin resulting from apoptosis. However, removal of APC in *Xenopus* extracts impairs apoptosis [20]. Consistently, we observed that in the absence of APC apoptotic fragmentation of chromatin and spindle disassembly was delayed by 1–2 hours compared to controls (not shown). Therefore, changes in the compaction of chromatin in extracts depleted of APC could not be attributed to apoptosis.

In addition, mitotic kinase activity of the CSF extracts was not significantly altered by APC depletion (Fig. S1), indicating that differences in chromatin compaction in the absence of APC are not caused by changes in cell cycle status.

This suggested that APC depletion affects the state of chromatin and that abnormal chromatin compaction could contribute to the weak spindle phenotype.

### APC Depletion Induces Over-compaction of Mitotic Chromatin in HeLa Cells

**Figure 2:**
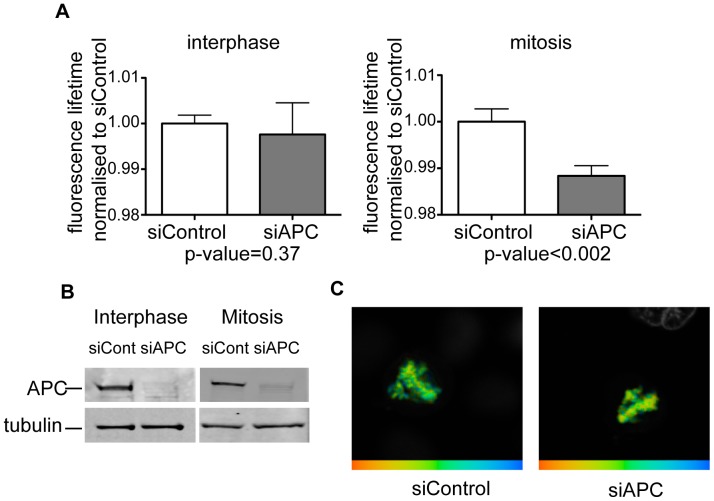
APC depletion increases chromatin compaction in HeLa cells. Asynchronously growing HeLa cells stably expressing GFP-H2B and mCherry-H2B were depleted of APC using siRNA (siAPC) or transfected with non-targeting siRNA (siContr), and donor fluorescence lifetime was measured using time-correlated single photon counting technique. ***A*** *.* Normalised lifetime values for APC- (grey bars) and mock- (white bars) depleted interphase cells (left) and mitotic cells (right). P-values are given underneath the plots. Please note that APC-depleted mitotic cells display significantly shorter FRET lifetime, indicating a higher degree of chromatin compaction. At least ten cells were measured for each condition. ***B*** *.* Level of APC depletion of cells used in A is visualized by immunobloting the corresponding lysates with anti-APC antibodies. Tubulin is used as a loading control. ***C*** *.* Representative chromatin images from control (left) and APC-depleted (right) mitotic cells. FRET efficiency is shown in false colors, with blue corresponding to low FRET efficiency (i.e. less compaction), and red corresponding to high FRET efficiency (i.e. more compaction).

In order to determine whether APC also affects chromatin morphology in mammalian cells, we measured the effect of APC removal on chromatin compaction in HeLa cells, using a recently described FLIM-FRET-based assay [13]. HeLa cells co-expressing GFP- and mCherry-labeled H2B histones were depleted of APC by siRNA or treated with non-targeting siRNA control (Fig. 2 B), and donor fluorescence lifetime was measured in interphase and mitotic cells in an asynchronously growing cell population (Fig. 2 A, C). Fluorescence lifetime in this system decreases with an increase in chromatin compaction due to increased FRET between labeled histones on separate nucleosomes that are brought into close proximity [13]. We found that APC loss increased the compaction of chromatin specifically in mitotic cells, while it did not affect the compaction of interphase chromatin (Fig. 2 A, C). Thus, lack of APC causes increased compaction of mitotic chromatin.

### Normal Proteome Composition of Sperm-derived Chromatin in Xenopus Egg Extract Requires APC

**Figure 3:**
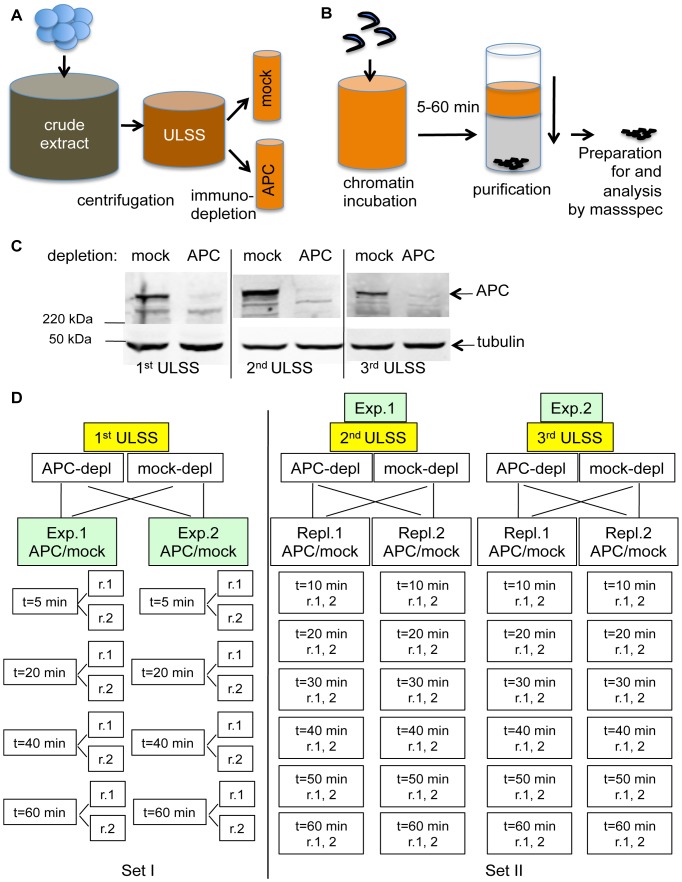
Experimental layout for proteomic analysis of chromatin-associated proteins in APC-depleted and mock-depleted *Xenopus* extract. ***A*** *.* Schematic representation of extract preparation. CSF (crude) extract obtained from *Xenopus* eggs was spun as in Methods, and a clear golden fraction was collected (ULSS extract). It was then incubated for 1 h with Dynabeads loaded either with affinity purified APC-specific antibody or non-specific rabbit IgG in the same concentration. Antibody-bound Dynabeads were magnetically retrieved, and the depleted extracts were used in the experiment. ***B*** *.* Schematic representation of chromatin manipulation in the experiment. Demembranated chromatin was added to the extract at concentration 20 ng per 1 µl of extract, mixed and incubated for indicated length of time (as in D.) before isolation by centrifugation through 30% sucrose cushion. After several washes chromatin was trypsinised and processed for mass spectrometry as described in Materials and Methods. C. Level of APC depletion in three extract preparations used in proteomics screen visualized by immunoblotting with anti-APC antibodies (APCII) and anti-tubulin antibodies as a loading control. 1 µl of corresponding extract were loaded into each lane and separated on PAGE prior immunoblotting. ***D*** *.* Experimental layout. In the 1^st^ set (left), mock- and APC-depleted 1^st^ ULSS extract were each separated into two halves and analysed as two technical replicas (exp. 1 and exp 2). Chromatin incubated for 5, 20, 40 or 60 min in these extracts was isolated as in B., and analysed by mass-spectrometry twice for each point (r.1 and r.2). In the 2^nd^ set, two independently derived and immunodepleted extracts (2^nd^ and 3^rd^ ULSS) were used in two identically performed experiments. Within each experiment, both APC and mock-depleted extract were separated into two halves (repl.1 and repl.2) and each was incubated with chromatin for 10, 20, 30, 40, 50 or 60 min. Chromatin associated peptides were measured with two different settings for each point, producing one extended measurement. (r.1, 2).

In order to gain an insight into the molecular causes of the increased chromatin compaction induced by APC depletion, we used a proteomic approach to identify changes in the composition of chromatin-associated protein complexes induced by APC depletion. We incubated sperm chromatin with *Xenopus* egg extract that was immunodepleted either with anti-APC or control rabbit antibodies, re-isolated the chromatin from the extract and analysed chromatin composition by quantitative, label-free mass spectrometry (Fig. 3 A, B, C). In this experiment we used CSF extract that was partially cleared by low speed centrifugation (ULSS extract), because it is more compatible with the experimental setup for mass-spectrometry [21]. Sperm-derived chromatin in ULSS extract underwent similar morphological changes to those observed in CSF extract, and APC depletion caused similar abnormalities in chromatin compaction in such extracts (Fig. 1H, I).

When placed in *Xenopus* egg extract, demembranated *Xenopus* sperm chromatin undergoes a nucleoplasmin-dependent remodeling process so that sperm-specific proteins are replaced by oocyte-derived histones, a process that is accompanied by transient chromatin decondensation [22]–[23]. However, changes are not restricted to histone-like molecules: the abundance of the majority of proteins on chromatin continues to change for at least 1.5 hours from a start of incubation with the interphase extract [21]. Therefore, in order to account for the dynamic nature of chromatin in *Xenopus* egg extract, we analysed the chromatin-bound material at several time points during spindle formation. Because spindle formation in this system is completed within an hour after addition of chromatin to the extract, we focused on chromatin changes during the first 60 min.

In the first experimental set, duplicate samples of sperm chromatin were incubated with APC- or mock-depleted extract for 5, 20, 40 and 60 min. (Fig. 3 D, Set I). Two further experiments were performed using two independently produced extracts (Fig. 3 D, Set II). After APC- or mock-depletion, duplicate samples of each extract were incubated with chromatin for 10, 20, 30, 40, 50 and 60 minutes before chromatin was isolated and analysed by mass spectrometry.

In total, just over 1000 proteins were quantified in two sets of experiments. Among those, 231 proteins displayed reproducible changes in their chromatin association in all experimental replicas. This dataset, although far from complete, was considered sufficiently reproducible to accurately reflect the dynamics of association (e.g. the temporal changes in abundance on chromatin throughout the time course) of chromatin binding factors. (Detailed description of data analysis is provided in the Material and Methods).

**Figure 4:**
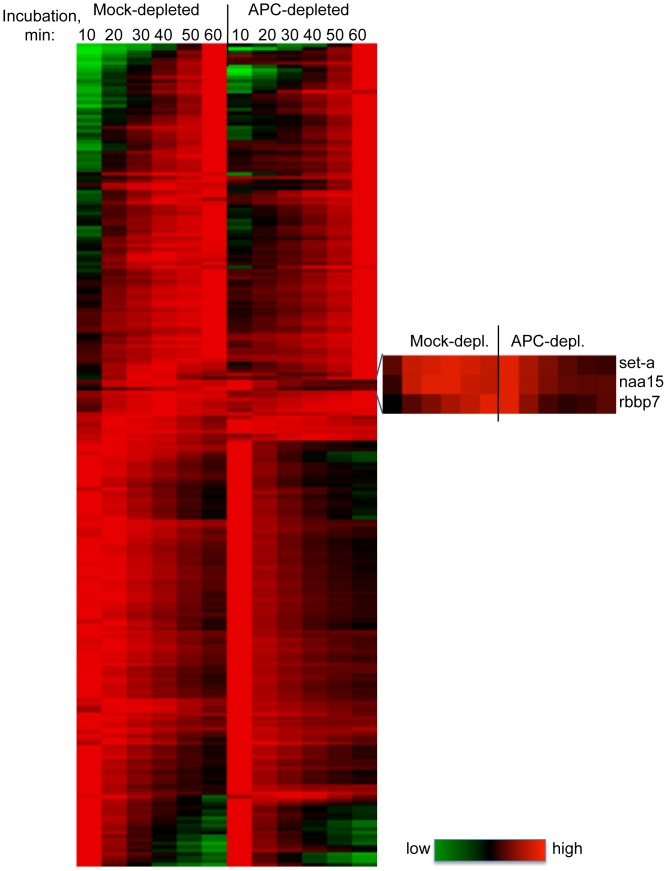
Temporal chromatin association profiles of proteins identified in proteomic screen in APC-depleted and control extracts. Data from 231 protein ID’s that display similar dynamics in all eight analyses (in three independent extracts, see Fig. 3 D) were clustered using TreeView, and displayed as a heat map. Green, black and red represent low, intermediate and high signal, respectively. An enlarged insert depicts a cluster of proteins with dynamics that vary significantly after APC depletion.

As shown in Fig. 4, the dynamics of chromatin association of most of these 231 proteins was generally similar between control and APC-depleted samples. However, a subset of proteins displayed different dynamics in control and APC-depleted samples (Table 1 and Fig. 4, insert). Three of them (rbbp7, set-a and smc6) are known chromatin regulators, and one (cdk1a) is a master regulator of mitosis. In addition, a glycolytic enzyme (pkm2), a subunit of the dynactin complex (actr1a), a potential regulator of myc and CCND2 translation (caprin1), and a protein of unknown function that contains RCC1 and BTB domains (rcbtb1) depend on APC for their normal association with mitotic chromatin.

**Table 1:** Proteins with altered chromatin association dynamics in the absence of APC.

name	ID	description	max pept Nr	P
rbbp7	160420243	histone-binding protein	8, 6/7, 6	−0.85
naa15	160420129	N(alpha)-acetyltransferase 15, NatA auxiliary subunit	4, 1/3, 7	−0.84
set-a	147900273	SET nuclear oncogen	10, 10/5, 6	−0.75
pkm2	148225037	pyruvate kinase muscle isozyme	62, 61/6, 6	−0.75
caprin1	147900243	cell cycle associated protein 1	5, 3/3, 7	−0.65
smc6	147907130	Structural maintenance of chromosomes protein 6	20, 27/2, 5	−0.43
actr1a	148226057	ARP1 actin-related protein 1 homolog A, centractin alpha	20, 23/7, 9	0.11
LOC446975	51968294		18, 14/5, 7	0.22
MGC52725	147898735		14, 16/6, 11	0.26
rcbtb1	148225636	regulator of chromosome condensation (RCC1) and BTB (POZ)domain containing protein 1	14, 11/2, 3	0.26
cdk1-a	148235959	cell division control protein 1-A	44, 41/24, 20	0.27

Data for proteins that were identified in both experiments, and showed reproducible dynamics within each experimental set, were averaged and smoothened to compensate for missing data. Similarity between APC-depleted and control dynamics was assessed using Pearson coefficient (P). Protein IDs that displayed dynamics that were different in APC-depleted extract from those in mock-depleted extract (Pearson coefficient below 0.3) are represented in the table. The maximum number of peptides detected in mock-depleted extract (set I), APC-depleted extract (set I)/mock-depleted extract (set II), APC-depleted extract (set II) are provided.

Also of interest were those proteins that bound chromatin in control and APC-depleted extract with similar dynamic trend, but were over- or underrepresented in the absence of APC throughout the time course (Tables 2 and 3 respectively). One transcriptional repressor (zhx3) and at least three proteins involved in translation (pcbp2, eif3h and mcts1-a) were elevated in the absence of APC, as was the kinetochore-associated checkpoint protein Bub3. On the other hand, the amount of two proteins involved in N-glycosylation (alg8 and ribophorin II), two microtubule associated proteins with known functions at kinetochores (dynactin 1 and CenpE) and a precursor of wntless homolog B, known to participate in sorting and secretion of Wnt proteins in *Drosophila*, were reduced on chromatin assembled in the absence of APC.

**Table 2:** Proteins elevated on chromatin in the absence of APC, but with unchanged dynamics of chromatin association.

name	ID	description	max pept Nr	fc up	fc up set 1	fc up set 2	P
MGC69154	148225843	hypothetical protein LOC379587	4, 8/1, 3	3.02	1.24	10.46	1.00
pcbp2	147901405	poly(rC) binding protein 2	7, 5/1, 4	2.02	1.20	3.80	0.62
eif3h	148225274	eukaryotic translation initiationfactor 3 subunit H	2, 3/2, 1	1.90	1.50	3.34	0.84
zhx3	148233976	zinc fingers and homeoboxes 3	14, 26/1, 2	1.79	1.37	3.41	1.00
txndc9	27924448	Apacd-prov protein	1, 2/3, 4	1.74	1.91	1.60	0.89
bub3	147900009	budding uninhibited bybenzimidazoles 3 homolog	2, 5/2, 2	1.64	1.66	1.41	0.88
mcts1-a	148236942	malignant T cell-amplified sequence1-A	3, 3/9, 11	1.54	1.52	1.95	0.75

The total amount of the signal for each protein ID in control or APC-depleted sample was calculated as a sum of the values in all time points from the smoothened profile, and averaged between the two sets. These values were further normalized to the total signal from all protein IDs in either control or APC-depleted samples in this dataset. The fold change increase (“fc up”) was calculated as a ratio between APC-depleted and control total values calculated as above. For individual sets, the fold change increase (“fc up set 1” and “fc up set 2”) was calculated from the raw data (not smoothened and not normalized to the total signal in the whole sample). Only data with a reproducible increase in the total signal in the absence of APC are presented. The positive Pearson coefficient (P) (P>0.3) given in the last row confirms similarity in dynamics of these proteins between APC-deficient and control samples. The maximum number of peptides detected in mock-depleted extract (set I), APC-depleted extract (set I)/mock-depleted extract (set II), APC-depleted extract (set II) are provided.

**Table 3:** Proteins reduced on chromatin in the absence of APC, but with unchanged dynamics of chromatin association.

name	ID	description	max pept Nr	fc down	fc down set 1	fc down set 2	P
wls-b	148228006	protein wntless homolog B precursor	4, 1/3, 2	2.83	1.24	3.39	0.47
dctn1	111305480	dctn1 protein	12, 9/1, 2	1.91	1.62	1.27	0.73
alg8	147901994	asparagine-linked glycosylation 8,alpha-1,3-glucosyltransferase homolog	13, 5/4, 3	1.88	2.44	1.09	0.93
rpn2	148235541	ribophorin II	17, 11/4, 3	1.87	2.1	1.1	0.95
cenpe	147900710	centromere protein E, 312 kDa	80, 44/5, 2	1.85	1.46	2.27	0.81

Data were processed as in table 2. The fold change decrease (“fc down”) was calculated as a ratio between control and APC-depleted total values calculated as above; the fold change down for individual sets, calculated from raw data are also given. Only data with a reproducible decrease in the total signal in the absence of APC are presented. The positive Pearson coefficient (P) (P>0.3) given in the last row confirms similarity in dynamics of these proteins between APC-deficient and control samples.

### APC Depletion has a Mild Effect on the Abundance of Topoisomerase II, Condensins, Kif4 and Linker Histones on Chromatin

Based on our findings that loss of APC caused an increase in chromatin compaction we first focused our attention on changes in known regulators of chromatin compaction. The major factors responsible for mitotic condensation are condensin I and condensin II, two related complexes that share common Structural Maintenance of Chromosomes (SMC) proteins but utilize different sets of accessory subunits [24]. Both complexes are found in *Xenopus* and participate in shaping mitotic chromosomes [25], [26]. Topoisomerase II is another prominent component of the axial scaffold of mitotic chromatids [27] that is required for mitotic chromatin condensation in several species [28]–[29]. In *Xenopus* egg extracts, Topoisomerase II is essential for condensation of exogenously added chicken erythrocyte nuclei [30]. Chromokinesin Kif4 has also been implicated in compaction of mitotic chromatin [31]. Finally, oocyte-specific linker histone B4 contributes to proper chromatin compaction in mitotic *Xenopus* egg extract, and a somatic linker histone H1 can also perform this function at high concentration [32]–[33].

**Figure 5:**
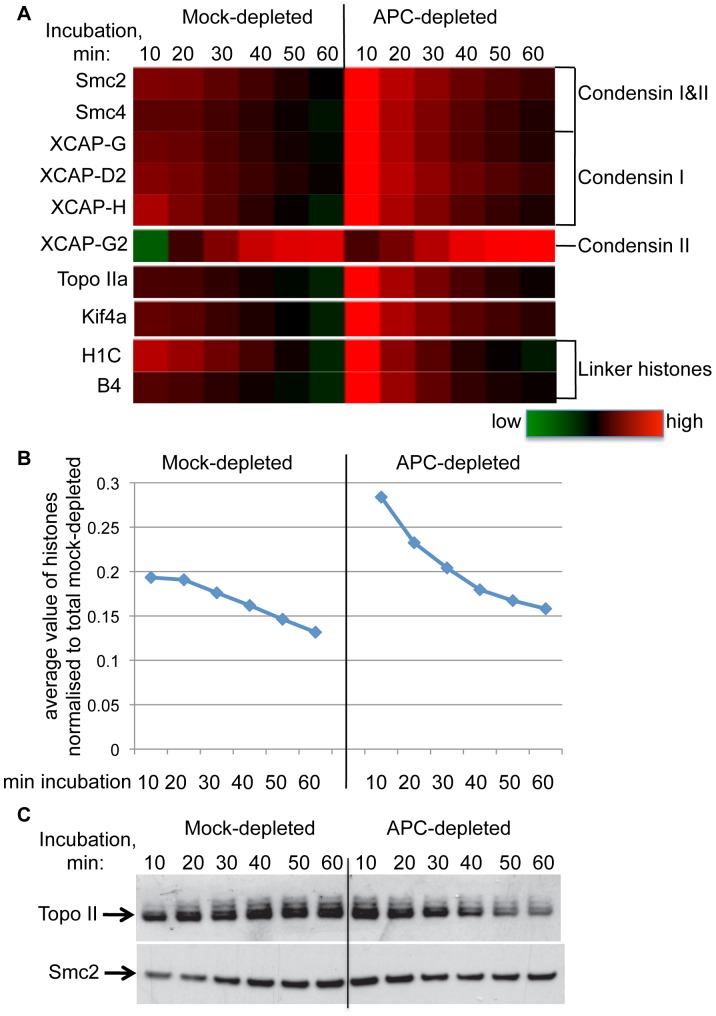
Effect of APC depletion on known mediators of chromatin compaction. ***A.*** Dynamics of chromatin association of indicated components of condensin I and II complexes, Topoisomerase IIa, Kif4 and two linker histones in mock-and APC-depleted extract, as determined in the proteomic screen is represented in a heat map, color-coded as in fig. 4. ***B*** *.* Average values from all histones identified in the screen (H2B, H2A, H2A.ZI2, H1C, hist1H4I and B4) normalized to corresponding total signal in mock-depleted samples. ***C*** *.* Immunoblotting of the chromatin samples prepared as for the proteomic screen, with antibodies recognizing *Xenopus* TopoII (top panel) and Smc2 (bottom panel). Each lane contains the equivalent of 200 ng chromatin.

Strikingly, all components of condensin I, as well as Topoisomerase IIa, Kif4 and both linker histones (somatic H1C and oocyte-specific B4) displayed similar profiles in their dynamics of chromatin association (Fig. 5 A). In control extract the amount of these proteins decreased slightly throughout the time course, while in APC-depleted extract the initial level was higher than in controls but then declined more rapidly. In contrast, condensin II subunit XCAP-G2 increased strongly on chromatin with time, both in control and APC-depleted extract (Fig. 5 A).

Furthermore, these changes closely paralleled changes in the dynamics of all histones identified in our proteomics screen (Fig. 5 B), so that the ratio between condensin I subunits or Topoisomerase IIa and the averaged signal from all identified histones remained constant, and was not affected by APC depletion (Fig. S2). Importantly, such pattern in the level of histones was observed in each individual experiment performed, indicating that this was not due to unequal sample volumes.

Despite the fact that the difference in dynamics and levels of these proteins on chromatin between control and APC–depleted extracts was below the thresholds set up for Tables 1, 2 and 3 (see Materials and Methods), we aimed to determine if these relatively subtle changes could be detected directly. Immunoblotting chromatin isolated after incubation with control or APC-depleted extract with anti-Topoisomerase II antibodies confirmed both the relative increase in the initial level, and the subsequent steep decline of Topoisomerase II on chromatin incubated in APC–depleted extract (Fig. 5 C). The level of Topoisomerase II on chromatin in control extracts appeared constant and even showed a slight increase over time. Similar changes were observed for Smc2 protein (Fig. 5 C). However, the decline of Smc2 levels on chromatin in the absence of APC was less pronounced than that of Topo II.

### APC Depletion Reduces Association of Set-a and Rbbp7 with Chromatin in CSF Extracts

**Figure 6:**
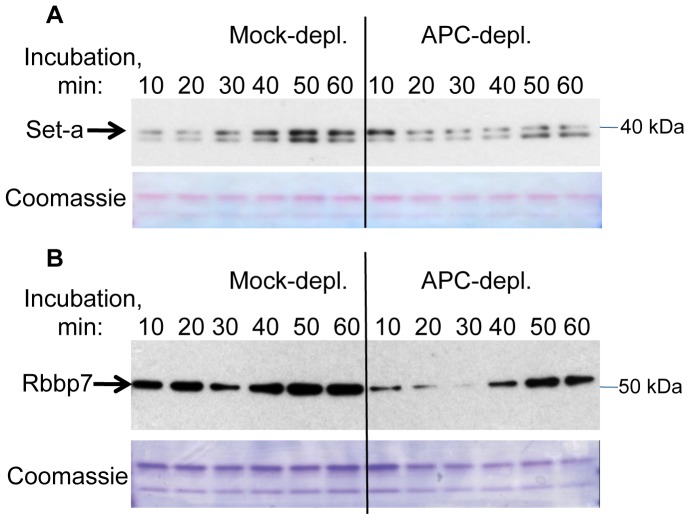
Effect of APC depletion on association of Set-a and Rbbp7 with chromatin. Immunoblotting of chromatin samples prepared as for proteomic screen, with antibodies against Set-a (A) and Rbbp7 (B), as well as the Coomassie staining of the bottom portions of the gel used as a loading control. Each lane contains the equivalent of 200 ng chromatin.

We aimed to further validate the changes we observed in APC–dependent chromatin association of selected candidate proteins identified in our proteomics screen, using immunoblotting. The lack of available antibodies that recognize *Xenopus* proteins prompted us to use antibodies specific for the respective human proteins. To detect *Xenopus* Set-a we used an anti-human Set (nuclear oncogen, TAF-Iβ) antibody (Bethyl Laboratories) produced against residues 175–220 of human Set, which are identical in *Xenopus* Set-a. This antibody recognised a doublet that migrated around 40 kDa (Fig. 6A), the predicted molecular weight for the *Xenopus* Set-a.

Immunoblotting of the chromatin isolated after incubation with APC-depleted or control extract with this antibody showed that the upper protein band perfectly mirrored the dynamics of Set-a protein detected in the proteomic screen: while in control extracts the protein gradually accumulated on chromatin with time, in APC-deficient extracts it was most abundant at the beginning of the time course (10 min) and then declined. The dynamic behavior of the lower molecular weight protein detected by this antibody was similar to that of Set-a in both types of extracts suggesting that this protein may represent another SET-related protein in *Xenopus* egg extract.

We also used anti-human Rbbp7 antibodies (Sigma) raised against a fragment of Rbbp7 shared between human and *Xenopus* (residues 323–375 of *Xenopus* Rbbp7), to validate the difference in the association of Rbbp7 with chromatin after APC depletion revealed by our proteomic screen. The antibody recognised a single band of approximately 52 kDa, the predicted size of *Xenopus* Rbbp7 (Fig. 6 B). The amount of Rbbp7 protein increased over time in the control samples, while in APC–depleted samples the Rbbp7 level initially declined and then increased again at later time points. This was similar to the dynamics of Rbbp7 detected in the proteomic screen. However, the immunoblot showed a reduction in Rbbp7 level in the control sample taken at 30 min of incubation, which was not apparent in the proteomic data. Therefore, the dynamic trend of Rbbp7 protein on the immunoblot appeared to be similar between control and APC-depleted extracts. The most notable difference in Rbbp7 in the absence of APC was an overall reduction in chromatin–associated Rbbp7 protein throughout the time course.

Together, these results show that APC affects the temporal association of Set-a, Rbbp7 and several other proteins with chromatin during spindle formation, consistent with the observed change in mitotic chromatin compaction.

## Discussion

A role for APC in maintaining chromosomal stability can be partially attributed to its effect on mitotic progression. APC depletion, or overexpression of a truncated APC in various models induces a plethora of mitotic defects ranging from centrosomal dysfunction, spindle destabilization, abnormally oriented kinetochores, cytokinetic failure to compromised spindle assembly checkpoint resulting in chromosome segregation errors [19] [9]–[10] [8] [7] [34]–[35]. Here we describe a novel role for APC in the compaction of mitotic chromatin.

We found similar effects of APC depletion on mitotic chromatin compaction in two distinct experimental systems: sperm chromatin remodeled in *Xenopus* egg extract and mammalian cells. Both systems revealed increased chromatin density as visualized by increased local DAPI fluorescence within a decreased volume of chromatin (Fig. 1) or a reduction in the donor fluorescence lifetime of neighbouring fluorescent histones in a FLIM-FRET-based assay, indicating shorter distance between histones (Fig. 2). Another indication of the change in chromatin compaction in APC-deficient *Xenopus* extract was the loss of fine threads of chromatin that normally stretch out from the bulk of fully condensed chromatin in mitotic *Xenopus* extract. These threads are likely to be small groups of chromatids that become individualised at a late stage of chromatin remodeling in mitotic extract [36]. The lack of these structures in the absence of APC could be the result of increased chromatin stiffness or may be due to increased axial shortening consistent with over-condensation. Alternatively, it could represent a failure to separate these structures from the rest of the chromatin, common in systems with insufficient chromatin condensation or Topoisomerase II activity [37].

Regardless of the complexity of such chromatin alteration, we observe a strong correlation between aberrant chromatin morphology and a “weak spindle” phenotype in the absence of APC, when comparing these parameters on a spindle-by-spindle basis (Fig. 1 D). It is plausible that abnormal chromatin compaction contributes to the loss of microtubule density in spindles in this system.

Many models for how chromatin compacts during mitosis have been proposed including radial loops, hierarchical folding, hierarchical folding with axial “glue” etc. ([38], and references therein). Nonetheless, exactly how chromatin folds in mitosis remains enigmatic [39]. It is therefore difficult to speculate what molecular changes could cause over-compaction of chromatin in mitosis. Published cases of over-compaction of chromatin usually refer to either an axial shortening of isolated chromatids [31], [40] or to premature chromatin condensation (PCC) leading to prophase-like chromosome compaction outside of mitosis [41]. None of these phenotypes mirror the gross compaction changes that we observe in these two APC-deficient systems.

Therefore, in the absence of mechanistic or phenotypical clues about the potential molecular mediator(s) of chromatin over-compaction induced by the absence of APC, we reasoned that such mediator is likely to be a chromatin-associated factor whose abundance on chromatin is dependent on APC. Thus our screen aimed to identify temporal changes in composition of the chromatin-associated proteome in *Xenopus* egg extract induced by APC depletion.

In particular, we found that the Set-a and Rbbp7 chromatin modifiers were underrepresented on chromatin in the absence of APC. SET/TAF-Iβ (alternative names for Set-a) was previously implicated in de-condensing sperm chromatin in *Xenopus* egg extract, [42] and human sperm nuclei *in vitro* [43]. In addition, SET was reported to regulate chromatin condensation in mammalian somatic cells: overexpression of SET in HeLa cells induced decondensation [43], and knocking down SET induced premature chromosome condensation (PCC) in mouse embryonic fibroblasts and condensed metaphases in human H1299 cells [44]. Thus, an excessive condensation of chromatin in our system could be attributed to the reduction in the level of chromatin-associated SET we detected.

What can cause such a reduction? In interphase, one of the SET-containing complexes, INHAT, binds to repressive (non-acetylated) chromatin marks and maintains chromatin in the closed, hypo-acetylated state by the activity of associated HDAC I or II [45], thus constituting a positive feedback loop. Although its functions in mitosis are not understood, it is conceivable that diminished SET association reflect a deficiency or a delay in establishing general transcriptional repression in APC-depleted chromatin. Reduced SET also could be secondary to mitotic state of the extract/chromatin. SET binds strongly to mitosis-specific S10 phosphorylated histone H3 [45], a known mitotic marker and a target of Aurora kinases [46]–[47].

Another possible reason for the aberrant chromatin condensation in *Xenopus* egg extracts could be a collective effect of small individual changes in the chromatin association of condensin I, Topoisomerase IIa, Kif4 and linker histones that mirrored the changes in the abundance of all histones identified in our screen. All of them were enriched on chromatin in APC-deficient extracts at the beginning of the time course. However, such enrichment was not sustained, and the levels of these factors decreased steeply with time. Interestingly, since the same trend was observed for all histones identified by our mass-spectrometry analysis, the primary effect of APC-depletion could be on the histone complement of chromatin itself.

In summary, we discovered a novel phenotypic trait induced by APC depletion, specifically over-condensation of mitotic chromatin, and show that at least in *Xenopus* egg extract, this is accompanied by temporal changes in the composition of specific chromatin and chromatin-associated regulators of chromatin compaction.

## Materials and Methods

### Chromatin

Demembranated *Xenopus* sperm nuclei were prepared as described [48], and resuspended in NBP (250 mM sucrose, 15 mM K-HEPES, pH 7.4, 1 mM EDTA, 0.5 mM spermidine trihydrochloride, 0.2 mM spermine tetrahydrochloride, 1 mM dithiothreitol [DTT], 10 µg/ml leupeptin) containing 0.3% bovine serum albumin and 30% (wt/vol) glycerol at a final concentration of up to 2 mg/ml (4.8×10^5^ sperm nuclei/ml).

### Extracts

CSF-arrested *Xenopus* egg extracts were prepared as described previously [49]–[50]. To prepare ULSS, de-gelled *Xenopus* eggs rinsed in UEB (50 mM KCl, 50 mM K-HEPES, pH = 7.6, 5 mM EGTA, 5 mM MgCl_2_, 2 mM DTT) with leupeptin, chymostatin and pepstatin A at 10 µg/ml final concentration, were crushed by 15 min centrifugation at 24.4 k g at 4°C in the presence of 100 µg/ml cytochalasin B. The cytoplasmic layer was collected and supplemented with 10 µg/ml cytochalasin D and 1/10^th^ volume of 2.5 mM ATP (up to 0.25 mM final concentraion) in LBF1–50 buffer (40 mM K-Hepes, pH = 8.0, 20 mM K-phosphate, pH = 8.0, 2 mM MgCl_2_, 1 mM EGTA, 2 mM DTT, 50 mM KCl, 10% sucrose) containing 0.5 mM PMSF and 10 µg/ml each leupeptin, chymostatin and pepstatin A, before further separation by 25 min centrifugation at 109 k×g at 4°C, to produce a clear golden supernatant (ULSS).

For APC immunodepletion, Dynabeads (Invitrogen) were loaded with affinity purified anti-APCII antibody [51] or rabbit purified IgG, and washed either in LBFI-50 (for ULSS extract) or in buffer containing 10 mM HEPES, pH = 7.7, 1 mM MgCl_2_, 100 mM KCl and 150 mM sucrose (for CSF extract), and incubated with appropriate extract for 1 hour at 4°C, with slow rotation. Dynabeads were then removed using a MPC-S magnet (Dynal), and depleted extracts were either snap-frozen (for ULSS) or used immediately in the experiment (for CSF extract). 100 µl of dynabeads with 25 µg of appropriate antibodies were used per 120 µl of CSF extract or per 60 µl of ULSS extract.

### Chromatin Reconstitution in Xenopus Egg Extracts

To assess chromatin morphology, demembranated sperm nuclei were incubated with CSF or ULSS extract for 40–65 min at room temperature, at 4–6×10^5^ nuclei per 1 µl of extract, prior spotting 2 µl of this mix onto a glass slide and fixing with 4% formaldehyde solution containing 60% glycerol, 1 µg/ml DAPI and 1×MMR buffer (100 mM NaCl, 2 mM KCl, 1 mM MgCl_2_, 2 mM CaCl_2_, 0.1 mM EGTA, 5 mM HEPES pH 7.8). For visualizing spindles, approximately 0.02 µg rhodamine-labelled bovine tubulin (Cytoskeleton) per 1 µl of extract (∼0.2 µM) were added simultaneously with chromatin. The samples were further examined using a 20×/0.7 NA lens on a Leica DMIRB microscope operated by Improvison Openlab 5.0 software. Images were captured with a Hamamatsu ORCA camera. Alternatively (for Fig. 1 H and I), 0.2 µm optical sections were collected throughout the entire thickness of chromatin using a Nikon-based DeltaVision deconvolution microscope with 40X PlanApo 1.30 NA lens, and sum intensity projections of deconvolved z-stacks were generated using Softworx®.

For the proteomics screen and samples for immunoblotting, sperm DNA was incubated with metaphase arrested (ULSS) extract supplemented with 7.5 mM creatine phosphate, 1 mM ATP, 1 mM MgCl_2_ and 0.25 mg/ml cyclohexamide for indicated time in the water bath at 23.5°C, at 20 ng DNA per 1 µl of ULSS. At the end of incubation the reaction mix was diluted six fold with NIB (50 mM KCl, 50 mM HEPES, pH = 7.6, 5 mM MgCl_2_, 2 mM DTT, 0.5 mM spermidine trihydrochloride and 0.15 mM spermine tetrahydrochloride) containing 10 µg/ml each leupeptin, chymostatin and pepstatin A, 1∶500 phosphatase inhibitor cocktail set II (Calbiochem), 0.1% Triton X100 and 2.5 mM ATP and spun through the cushion of 30% sucrose in NIB with 0.1% Triton X100, at 3000 g for 7 min at 4°C. Sucrose-buffer interphase was then wash once with XBEII (10 mM K-Hepes, pH = 7,7, 50 mM Sucrose, 100 mM KCl, 2 mM MgCl_2_, 100 mM CaCl_2_, 5 mM K-EGTA) containing 0.1%NP40, and once with XBEII, before aspirating the Sucrose layer. For immunoblotting, drained chromatin-containing pellets were directly resuspended in protein loading buffer 1×LSB (Invitrogen), supplemented with 50 mM DTT. For mass-spectrometry, chromatin pellet was propagated using filter-aided sample preparation (FASP) method exactly as described [52]. Tryptic peptides were desalted on homemade C18 Stage Tips [53]. LC-MS analysis was performed at Proteomics Facility of the Wellcome Trust Biocentre (University of Dundee, Dundee, Scotland, UK) using Thermo Fisher Scientific Orbitrap Velos mass spectrometers with Dionex nano-LC inlet system.

Peptides were eluted into mass spectrometer from reverse phase column with three step gradient 3–17% −90 min, 17–35% −22 min, 35–90% −15 min of buffer B (90% acetonitrile, 0.1% formic acid in H_2_O). Mass spectra were collected in data-depended mode. The 12 most intense ions above 500 counts threshold were sequentially isolated for MSMS analysis and were dynamically excluded from repeat acquisition for 135 sec. Combined peak lists were extracted from raw files by Maxquant 1.1.1.13 [54] and corresponding peptides were identified using Mascot program (Matrix Science, London, United Kingdom) against *Xenopus laevis* protein database (www.xenbase.org date stamp 07/05/2010) supplemented with common contaminants.

### Analysis of Proteomic Data

The label-free peptide quantification provided by Maxquant at the 1% FDR was then processed into protein profiles using in house written software “Chronoprot” that will be described in details elsewhere. Briefly, Chronoprot uses as an input the evidence file from Maxquant. It first assembles a time course profile for each peptide. To equalize the contribution of peptides to final protein profile, the average intensity of each peptide profiles for a given protein is normalized to the average intensity of three peptides with highest intensities. The protein profile is calculated as the average or the mean of its normalized peptides profiles. The final (optional) step is the smoothing of temporal profiles.

The smoothing procedure for protein profiles was implemented to decrease the influence of variations or absent values in processing of the samples. For smoothing we used the weighted average of 5 neighboring time points according to formula.
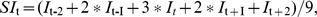
where *SI*_t_ – smoothed intensity in time point (t), *I*_t_ – original intensity in time point (t).

For the calculations of the border values, profiles were artificially extended by two time points in each direction with assigned values equaled to the first or the last time point correspondingly.

For quality control and reproducibility of the LC-MS procedure, equal volume aliquots from all experimental samples prepared for masspectrometry were mixed together and subdivided into four identical samples, which were analysed by LC-MS as the first and last sample in each experimental time series. Two additional controls were included in each experiment: 1) sperm incubated in buffer instead of extract and 2) extract without addition of sperm DNA. Both were incubated at 23.5°C for 60 min and processed for mass spec analysis exactly as experimental samples. The average variability between the quality control replicas was found to be 0.22 of the mean of the signal.

All temporal profiles and control samples in each of two experiments were quantified using the same peptides normalization allowing quantitative comparisons between all experimental conditions.

For the following analysis we used only proteins that had been identified in the apex of profile with 2 or more peptides.

The first experimental set yielded 1,047 protein IDs, of which 742 proteins were identified in both replicas in chromatin incubated with control extract and 748 proteins - in both APC-depleted time courses. Out of these, 79% proteins in control extract and 88% proteins in APC-depleted extract displayed reproducible dynamics between replicas, Pearson coefficient P>0.3. When a more stringent criteria was applied (P>0.5), reproducible dynamics of association was detected in 74% and 86% of proteins in control and APC-depleted extract, respectively.

The second experimental data set yielded 921 protein IDs, of which 482 (in control extracts) and 490 (in APC-depleted extracts) proteins were identified in both experiments. The reproducibility in the dynamics between two separate experiments in the second set was 47% for control and 53% for APC-depleted samples (Pearson coefficient P>0.3); at the more stringent P>0.5, 40% control and 46% APC-depleted samples showed dynamics reproducible between experiments. The lower reproducibility in this set was expected, as it compared chromatin binding proteins in two distinct extract preparations, while in first set we compared dynamic behavior of two experiments utilizing the same extracts.

In control preparations, 248 proteins were identified in a sample prepared from chromatin incubated with buffer instead of extract, and 153 proteins - in a sample that contained no chromatin. If the protein identified in the experiment was present in controls, the highest of the two measurements (chromatin only or extract only) was used as background. For these proteins the experimental data were manually inspected to ensure that the values analysed were at least 0.66 of the mean (three times variability) above background.

### H1 Kinase Assay

Aliquots of extract diluted 75 fold in buffer containing 80 mM Sodium beta glycerophosphate (pH = 7.3), 10 mM MgCl_2_, 5 mM EGTA, 1 mM Sodium orthovanadate, 1 mM DTT, 0.5 mM PMSF and leupeptin, chymostatin and pepstatin at a final concentration of 10 µg/ml were incubated for 10 min with equal volumes of substrate mix containing 40 mM HEPES (pH = 7.4), 20 mM MgCl_2_, 10 mM EGTA, 0.4 mg/ml Histone H1 (Roche) and 0.4 mM ATP (0.45 µCi/nmol) at room temperature before spotting the reaction onto P81 phosphocellulose paper, washing in 1% phosphoric acid, and measuring the radioactive signal. After subtracting the background that was measured in the same way but without Histone H1, the H1 kinase activity was calculated as number of incorporated phosphates per min of incubation per µl of extract, using the total signal of buffer containing 800 pmol ATP mix used in this assay for calibration.

### Western Blotting

CSF or ULSS extract (1 µl per line) or Hela cell lysed in MEBC buffer (50 mM Tris-HCl, pH 7.5, 100 mM NaCl, 5 mM EGTA, 5 mM EDTA, 0.5% NP-40, and 40 mM β-glycerol phosphate) were separated on 4–12% SDS PAGE (Invitrogen), transferred to Protran nitrocellulose membrane (Schleicher & Schuell) and immunoblotted with the following primary antibodies: anti-APCII anti-serum [51] (1∶1000), DM1A (Sigma; 1∶2000), rabbit polyclonal anti- *Xenopus* Topo II antbodies (1∶1000), sheep polyclonal anti-*Xenopus* Smc2 ([55], 1∶500), anti-human Rbbp7 (Sigma, 1∶500) and anti-human SET (nuclear oncogene) (Bethyl Laboratories, 1∶500). Secondary antibodies used were either horseradish peroxidase-labelled anti-rabbit IgG that were detected using ECL kit (Pierce), or IRDye800/700-conjugated secondary antibodies (Rockland, 1∶5000), detected with LiCor Odyssey Imager (Bioscience).

### HeLa siRNA

HeLa cells were cultured in DMEM (Gibco) supplemented with 10% FBS (PAA Laboratories), 1% penicillin-streptavidin stock solution (MP Biomedicals) and 1∶100 non-essential amino acids (Sigma-Aldrich). For siRNA-mediated APC protein or mock depletion, cells were transfected with 10 nM of appropriate non-targeting or APC-targeting siRNAs (Dharmacon) using Interferrin (Polyplus) according to the manufacturer’s instructions.

### FLIM-FRET Analysis

FLIM-FRET experiments were carried out on a HeLa H2b-2FP cell line [13] stably expressing GFP and mCherry tagged histone H2B as previously described by Lleres *et al.,* [*13*]. Cells were cultured in supplemented Leibovitz medium for the duration of imaging. Fluorescence Lifetime Imaging Microscopy (FLIM) was performed using an inverted laser scanning multiphoton microscope Radiance 2100MP (Bio-Rad Laboratories) equipped with temperature-controlled environmental chamber constructed with black walls to exclude external sources of light during the sensitive period of FLIM measurement. Measurements were acquired at 37°C, with a 60× oil immersion lens (1.4 NA). Two-photon excitation was achieved using a Chameleon Verdi-pumped ultrafast tunable (720–930 nm) laser (Coherent) to pump a mode-locked frequency-doubled Ti:Sapphire laser that provided sub-200-femtosecond pulses at a 90-Mhz repetition rate with an output power of 1.4 W at the peak of the tuning curve (800 nm). Enhanced detection of the scattered component of the emitted (fluorescence) photons was afforded by the use of fast single-photon response (5783P; Hamamatsu Photonics) direct detectors. The fluorescence lifetime imaging capability was provided by TCSPC electronics (SPC- 830; Becker & Hickl GmbH). TCSPC measures the time elapsed between laser pulses and the fluorescence photons. Fluorescence lifetime measurements were acquired over 60 s and fluorescence lifetimes were calculated for all pixels in the field of view (256×256 pixels) or for a particular selected region of interest (e.g., nucleus) using SPCImage software (Becker & Hickl, GmbH).

## Supporting Information

Figure S1 **Activity of mitotic kinase is not affected by APC depletion.** APC-depleted or mock-depleted CSF extracts supplemented with demembranated sperm chromatin were incubated at room temperature, and 2 µl aliquots were collected for kinase assay at indicated times. Kinase activity of the extracts towards recombinant histone H1 is plotted against time of chromatin incubation with the extract. Unit equals pmol of incorporated phosphate per min of kinase reaction.
(TIF)

Figure S2 **APC depletion does not affect the ratio of condensins and Topoisomerase II to histones.** Temporal profile of indicated proteins on chromatin incubated in mock- or APC-depleted extract determined in the proteomic screen, normalized to the corresponding average values of all identified histones (depicted in Fig. 5 B).
(TIF)
