# Supplementary figures and images for: The Adenomatous Polyposis Coli Protein Contributes to Normal Compaction of Mitotic Chromatin
Source: PLoS One. 2012 Jun 13;7(6):e38102. doi: 10.1371/journal.pone.0038102 (PMC3374815; doi:10.1371/journal.pone.0038102)

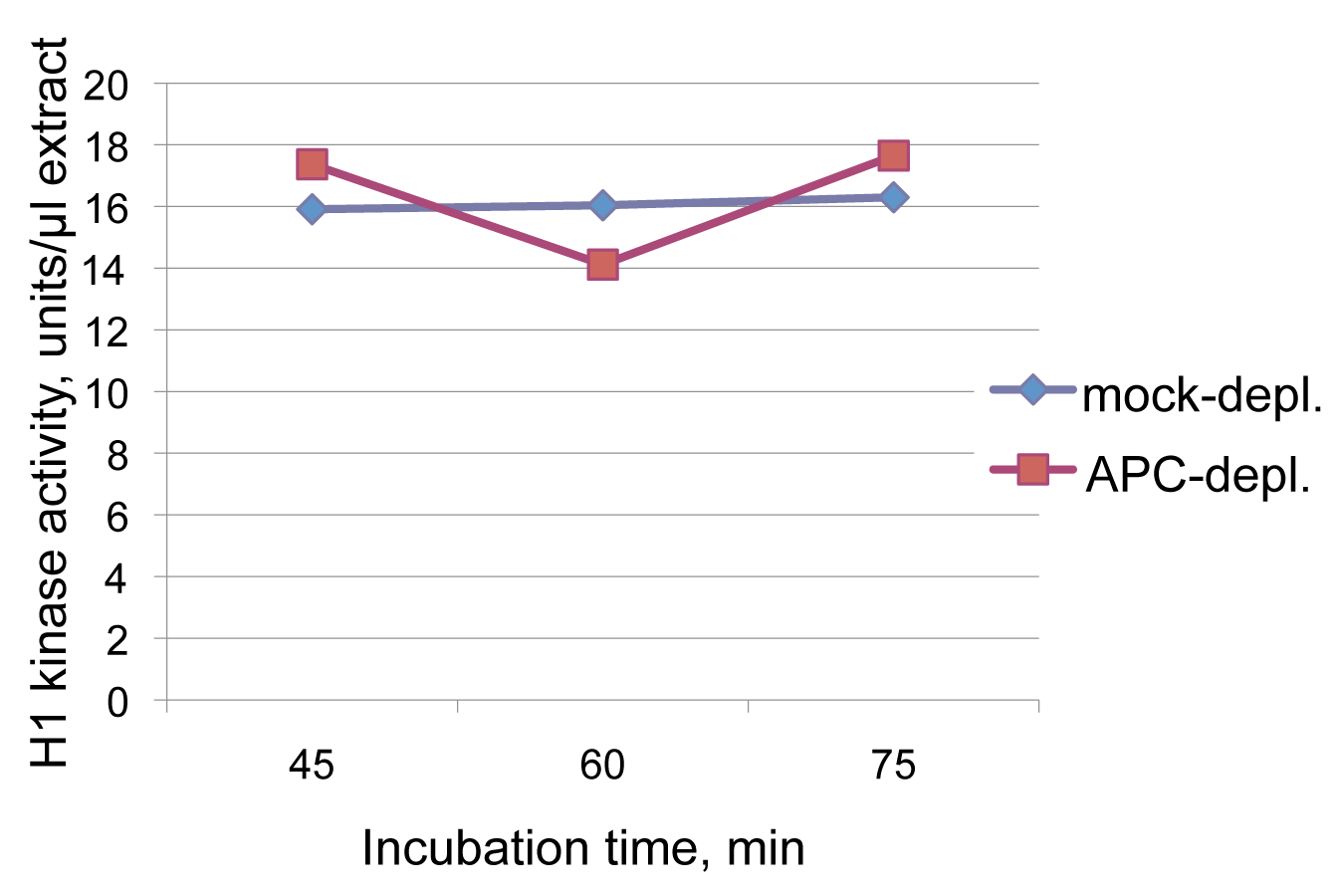

Supplement: Figure S1 — Activity of mitotic kinase is not affected by APC depletion. APC-depleted or mock-depleted CSF extracts supplemented with demembranated sperm chromatin were incubated at room temperature, and 2 µl aliquots were collected for kinase assay at indicated times. Kinase activity of the extracts towards recombinant histone H1 is plotted against time of chromatin incubation with the extract. Unit equals pmol of incorporated phosphate per min of kinase reaction. (TIF) [file pone.0038102.s001.tif]

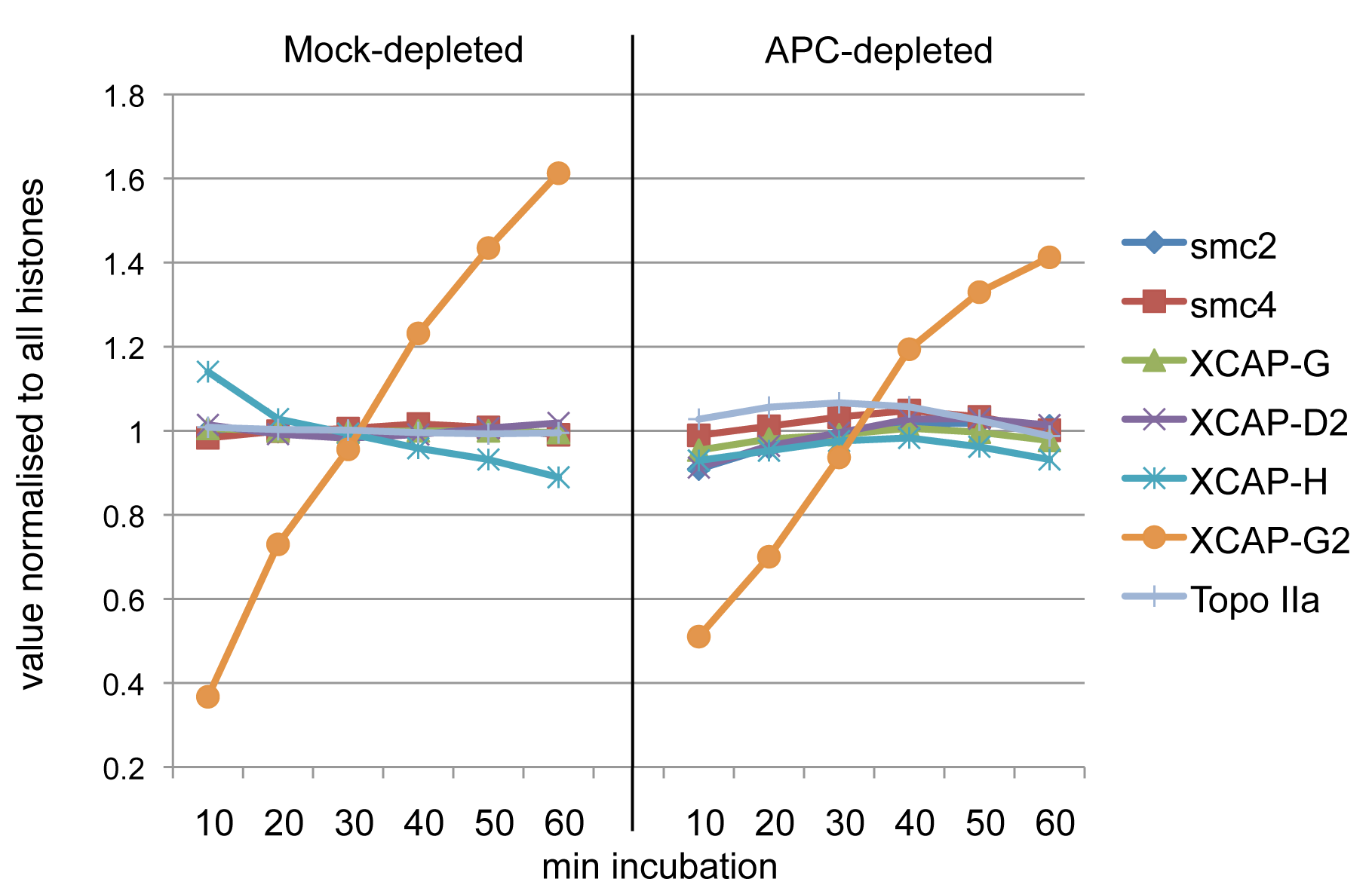

Supplement: Figure S2 — APC depletion does not affect the ratio of condensins and Topoisomerase II to histones. Temporal profile of indicated proteins on chromatin incubated in mock- or APC-depleted extract determined in the proteomic screen, normalized to the corresponding average values of all identified histones (depicted in Fig. 5 B). (TIF) [file pone.0038102.s002.tif]
